# Effect of Scanning Routes on the Stress and Deformation of Overhang Structures Fabricated by SLM

**DOI:** 10.3390/ma12010047

**Published:** 2018-12-24

**Authors:** Xiaochuan Zhang, Jinwu Kang, Yiming Rong, Pengyue Wu, Tao Feng

**Affiliations:** 1 Department of Mechanical Engineering, Tsinghua University, Beijing 100084, China; zhang-xc16@mails.tsinghua.edu.cn (X.Z.); rongym@sustc.edu.cn (Y.R.); 2 School of Materials Science and Engineering, Key Laboratory for Advanced Materials Processing Technology, Tsinghua University, Beijing 100084, China; 3 Department of Mechanical and Energy Engineering, Southern University of Science and Technology, Shenzhen 518055, China; 4 Beijing e-Plus 3D Tech. Co. Ltd., Beijing 100027, China; wupengyue@eplus3d.com (P.W.); fengtao@eplus3d.com (T.F.)

**Keywords:** SLM, overhang, scanning route, residual stress, deformation

## Abstract

Selective laser melting (SLM) is a promising manufacturing method for the construction of complicated precision parts. However, deformation of the overhang during the fabrication process and post treatment is still a common problem. In this paper, the effect of the scanning route on the residual stress and deformation of fabricated AlSi10Mg overhang specimens by SLM was investigated. Different scanning routes for the overhang including longitudinal direction, transverse direction, and the alternation between these two scanning routes in consecutive layers were studied by experiments within this study. Numerical simulation was utilized to measure the stress of the specimens while deformation prediction was used for the different scanning routes. Both the experimental and simulated results showed that the scanning route had a substantial influence on the residual stress and deformation of the specimens. The longitudinal scanning resulted in significant upward bending deformation of the overhang as it was cut from the baseplate. However, there was less deformation for the overhangs fabricated by transverse and alternating scanning routes. A transverse scanning route is helpful for the reduction of residual stress in the longitudinal direction and the corresponding deformation.

## 1. Introduction

Selective laser melting (SLM) is a 3D printing method for the fabrication of metal parts and is a promising fabrication method for the creation of complicated precision parts [1,2]. Many researchers have focused on the relationship between the fabrication technological parameters and porosity, and microstructure and mechanical properties [3,4,5,6,7,8,9,10,11]. On the other hand, the high cooling speed and large temperature gradient result in substantial residual stress and deformation during the fabrication process and post-treatment of the parts. Buchbinder et al. [12] studied the effects of baseplate preheating on the deformation of fabricated parts and found that raising preheating temperatures could reduce residual stress. Mugwagwa et al. [13] and Mercelis et al. [14] investigated the effects of fabrication parameters, scanning strategies, and laser power on the residual stress. In general, the stresses are larger perpendicular to the scanning direction than along the parallel scanning direction. The overhang structure is typically susceptible to deformation during the fabrication process and the post-treatment of the printed parts [14,15,16]. During the printing process the deformation of the overhangs can ruin the fabrication process. As the parts are cut from the baseplate the residual stress can result in significant deformation or cracks. The deformation of the printed parts is affected by their thermal history and their structural features of low stiffness. Some researchers have focused on the effects of the support on the deformation of the overhang structure [14,15,16]. The overhang structure results in slow cooling because of the inferior thermal conductivity of powder. The addition of supports, placed under the overhang structure, can facilitate its heat release and serve as constraints. The temperature distribution of the overhang structure is also influenced by the scanning route. Therefore, researchers have focused on the effect of the scanning route on the deformation of the overhang structure [15,16,17,18,19,20]. Michael et al. [15] and Kruth et al. [16] investigated the effect of the scanning route on the deformation of 316L alloy and tool steel parts, respectively, and hypothesized that a short scanning route is helpful in the reduction of deformation. Parry et al. [17] discovered that the residual stress in the scanning direction increases as the scanning length increases by numerical simulation results. Cheng et al. [18] believed that the rotation of the scanning direction by 45° for the consecutive layer was beneficial in the reduction of residual stress. Papadakis [19] and Neugebauer [20] simulated the deformation of the SLM-made stainless steel and In718 parts, respectively, after being cut from the baseplate but the temperature and stress fields were not provided. Although there is some research on the effects of scanning routes on the deformation of parts, there is no joint investigation of temperature distribution, stress, and the deformation of the entire process.

In this paper, the effects of different scanning routes on the deformation of an overhang feature made from AlSi10Mg alloy were systematically investigated.

## 2. Materials and Methods

**Figure 1:**
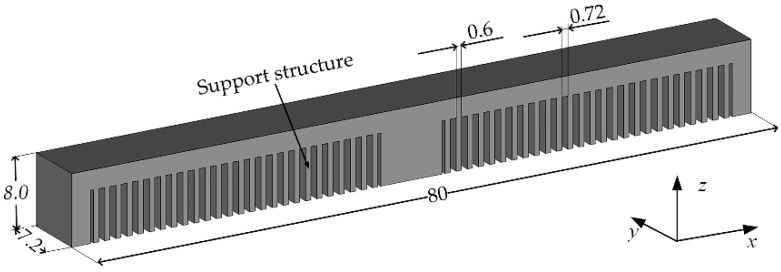
Schematic of an overhang specimen.

**Figure 2:**
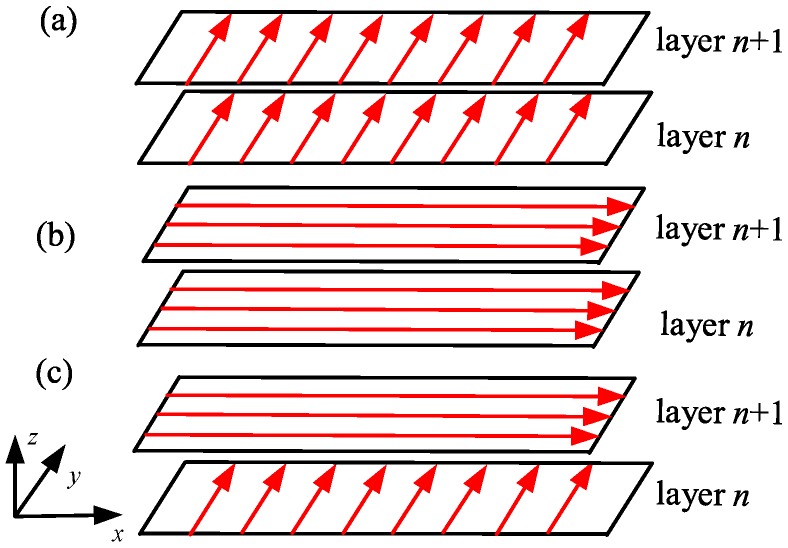
The different scanning strategies used: (**a**) scanning along the *y* direction (transverse scanning); (**b**) scanning along the *x* direction (longitudinal scanning); and, (**c**) alternative scanning along the *x* and *y* directions in consecutive layers.

**Figure 3:**
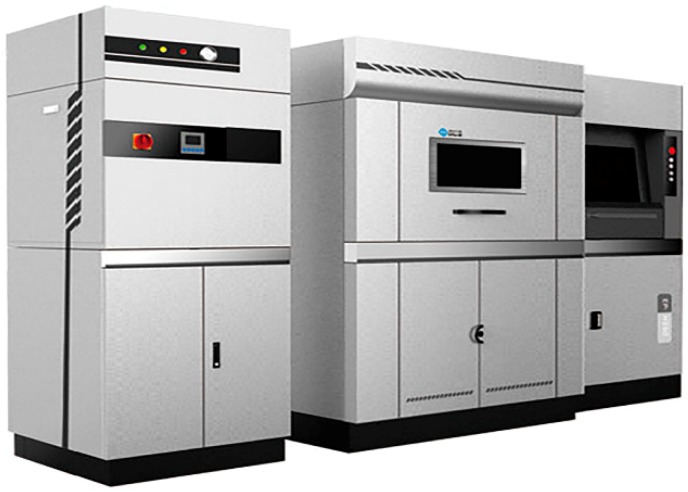
3D printer EP-M250 machine.

A symmetrical overhang structure with a size of 80 mm (length) × 7.2 mm (width) × 8.0 mm (height) was designed, as shown in Figure 1. The AlSi10Mg alloy powder used in this study was provided by AMC Technology Co. Ltd. (Beijing, China), the composition of which is listed in Table 1. A powder with the size of 20–60 μm was used. Three overhang specimens were fabricated using three different scanning strategies: (**a**) scanning along the *y* direction; (**b**) scanning along the *x* direction; and, (**c**) alternative scanning along the *x* and *y* directions, as shown in Figure 2. This was achieved using the 3D printer EP-M250, manufactured by Beijing e-Plus 3D Tech. Co. Ltd. (Beijing, China), as shown in Figure 3. The scanning speed was 1000 mm/s, laser power was 300 W, hatch space was 100 μm, and layer thickness was 30 μm. The baseplate was heated and kept at 25 °C. A series of slice supports parallel to the *y* direction were designed for the overhang at 0.6 mm thickness and at intervals of 0.72 mm apart. The specimens were then cut from the baseplate by electrical discharge machining. The deformation of the specimens was measured by a micrometer.

## 3. Results

**Figure 4:**
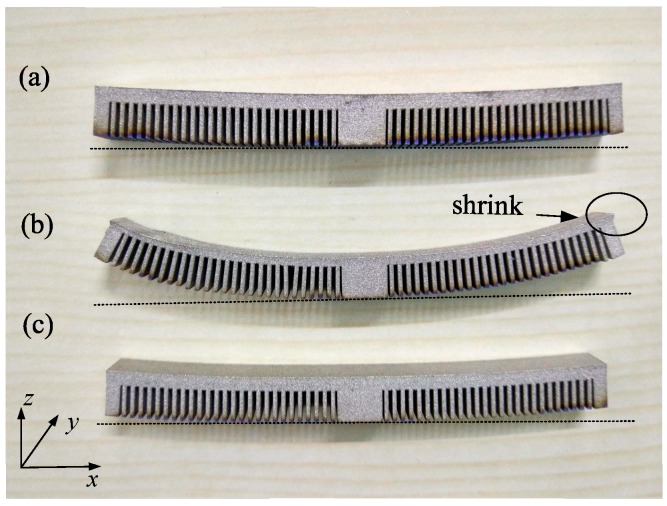
Deformation of the specimens with different scanning routes after being cut from the baseplate: (**a**) scanning along the *y* direction (transverse scanning); (**b**) scanning along the *x* direction (longitudinal scanning); and, (**c**) alternative Scanning along *x* and *y* direction in consecutive layers.

**Figure 5:**
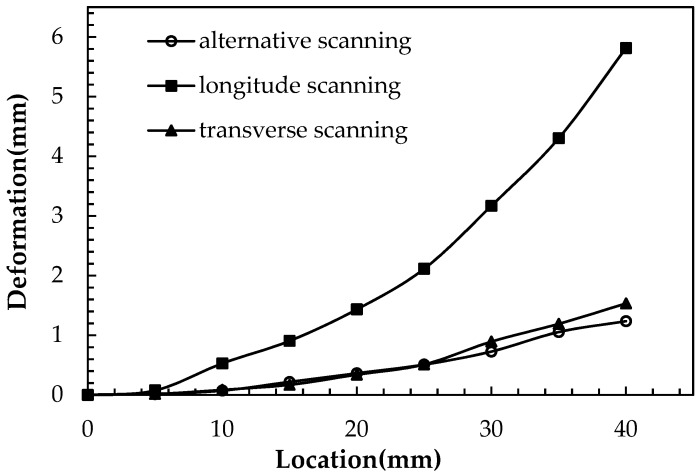
Deformation of the specimens with different scanning routes varying with the *x* axis.

As the overhang specimens were cut from the baseplate, different deformations were observed in the three specimens. There was significant upward bending deformation in the specimen scanned along the *x* direction reaching 5.8 mm in the *z* direction, while specimens scanned along the *y* direction, and the *x* and *y* directions were 1.5 mm and 1.4 mm, respectively, as shown in Figure 4. The deformation of the specimens varying with the *x* axis are plotted in Figure 5. As shown in Figure 4, the deformation increased from the center point to the ends in an essentially symmetrical manner. As marked in Figure 4, the corner shrinkage of specimen b, by scanning along the x direction, was significant while the shrinkage of specimens a and b, via the alternative scanning strategies, is hardly observable.

## 4. Analysis and Discussion

### 4.1. Numerical Simulation

**Figure 6:**
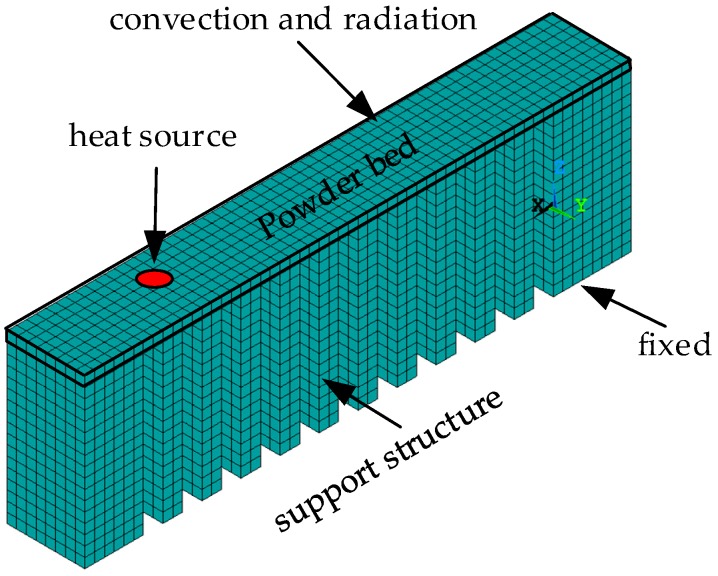
The FEM model and boundary conditions.

To analyze the effect of different scanning routes on the deformation of the specimens, thermo-stress coupled numerical simulation was performed using the software ANSYS 19.0. The non-linear thermo-elastoplastic model was adopted. As the tiny laser spot and layer thickness were far less than 1 mm, only a small area in several square millimeters was simulated [21,22]. Some researchers managed to simulate macroscopic scale geometries by the simplification of constitution models or by coarsening algorithms, but errors were introduced [23,24]. In this paper, the specimen was reduced to 1/10 for numerical simulation, to 2.8 mm (length) × 0.4 mm (width) × 0.9 mm (height), as shown in Figure 6. The specimen was meshed into cubes of 0.025 mm (length) × 0.025 mm (width) × 0.025 mm (thickness). The volume heat source was adopted as determined by Equation (1).

(1) $$q=\frac{Ap}{d_{c}d_{m}h}$$

where *q* is the heat flux, *A* is the absorption rate of 0.09 because of the high reflectivity, *d_c_* is the diameter of the laser spot 0.1 mm, *d_m_* is the layer thickness, and *h* is the hatch distance. The heat convection and radiation on the surface were treated as an equivalent heat transfer coefficient, varying with temperature. The thermal and mechanical parameters of the AlSi10Mg alloy used for the numerical simulations are listed in Table 2. Considering the latent heat for the phase change, the latent heat was 3.9 × 10^5^ J/kg.

The two ends of the Finite Element Method (FEM) model and the support structures should be finished first and set as solid states. Their initial temperature is 25 °C, room temperature. The overhang layer is initially set as powder at room temperature. As the laser point scans the powder bed, the powder volume covering is heated up, melted, and then turned into a solid state via the heat generated by the laser. As one layer of the overhang is fabricated, it is then cooled down to room temperature. The temperature fields then serve as the load input for stress analysis. The bottom of both ends and the supports are fixed in the *x*, *y* and *z* directions. As the stress analysis is performed for the fabrication process and the cooling process, the constraints at both ends of the parts and supports are removed and the static stress analysis is performed, which simulates the stress relief phenomena by the cutting of the parts from the baseplate and provides the final deformation.

### 4.2. Discussion

**Figure 7:**
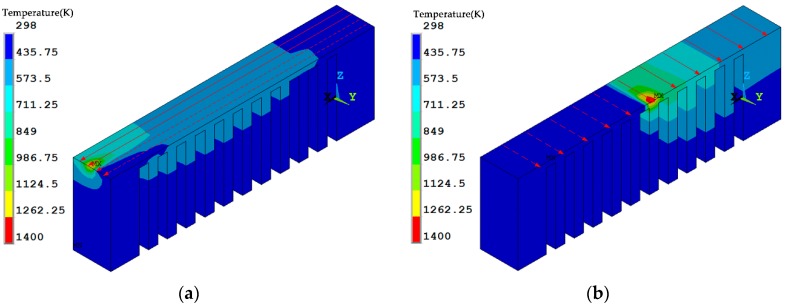
Temperature distribution under different scanning strategies during the printing process (solid vectors are the scanned tracks while the dashed vectors are to become scanned tracks): (**a**) the *x* scanning route; and, (**b**) the *y* scanning route.

**Figure 8:**
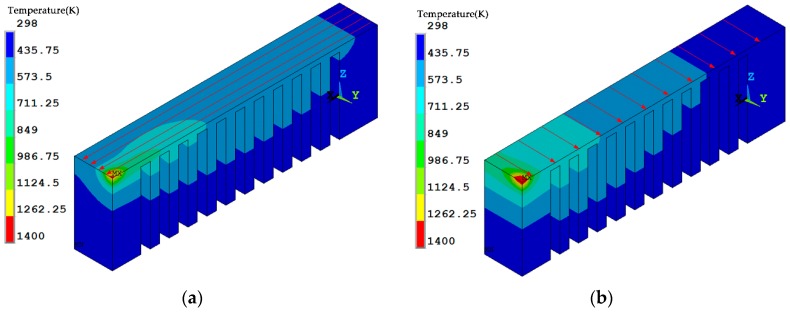
Temperature distribution under different scanning strategies at the end of laser scanning: (**a**) the *x* scanning route; and, (**b**) the *y* scanning route.

**Figure 9:**
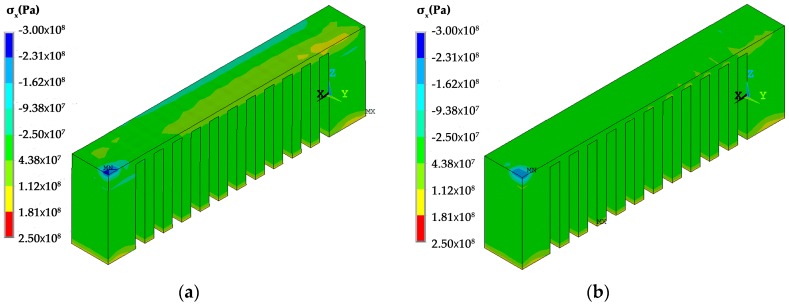
Residual stress σ_x_ distribution of the specimens under different scanning strategies at the end of laser scanning: (**a**) the *x* scanning route; and, (**b**) the *y* scanning route.

**Figure 10:**
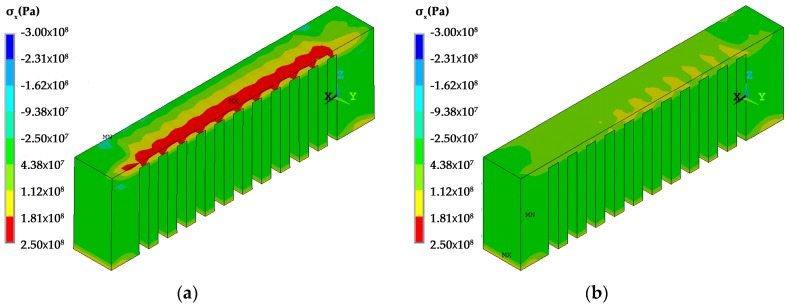
Residual stress σ_x_ distribution of the specimens under different scanning strategies as they are cooled to room temperature: (**a**) the *x* scanning route; and, (**b**) the *y* scanning route.

**Figure 11:**
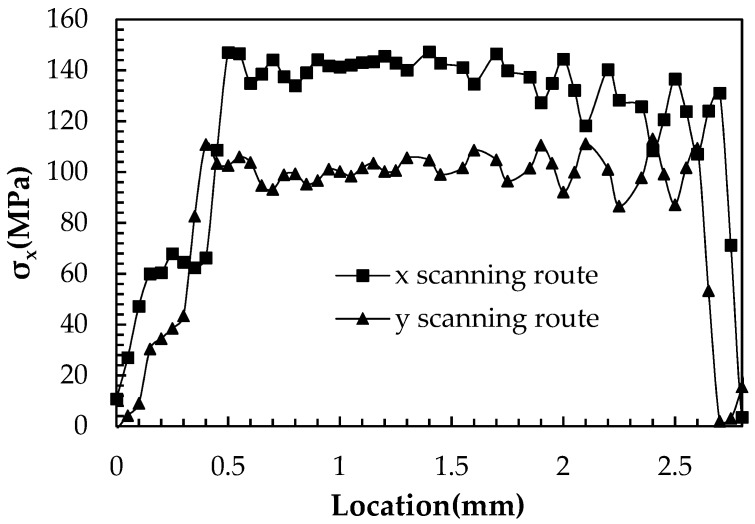
Residual stress σ_x_ distribution of the specimens under different scanning strategies as cooled to room temperature along the *X* path.

The temperature distribution during the laser scanning process under the two scanning routes is shown in Figure 7. The current scanning track heats up the neighboring fabricated area. Regarding the *x* scanning route, the current track heats up the entire length of the overhang but for the *y* scanning route the current track purely heats up a limited region of the overhang. The temperature distribution at the end of the laser scanning of the overhang layer is shown in Figure 8. The high temperature zone of the *x* scanning route is longer than the *y* scanning route because every track runs between the two ends under the condition of the *x* scanning route. For the *y* scanning route, each track purely affects the nearby areas with a slight effect on the starting end. Thus, as the laser scanning process finishes, the starting end has completely cooled down. The shrinkage of the whole overhang along the *x* direction roughly occurs at the same time as the former scanning route, while the shrinkage is only limited to the right half of the overhang for the latter scanning route. Due to the constraints of the baseplate, the shrinkage tendency difference determines their residual stress within the overhang layer. The residual stresses of the constraints of the baseplate along the *x* direction are tensile for both scanning strategies at the end of the laser scanning as well as room temperature, as shown in Figure 9, Figure 10 and Figure 11. The values for the former scanning are greater than the latter. As the specimen cools to room temperature the residual stress increases but the specimen remains in a tensile state. Under both conditions, the tensile residual stress along the x direction tends to bend the overhang layer upward as constraints are removed. 

The residual stress of the overhang structure is directly determined by its thermal history during the fabrication process. On the other hand, the microstructure of the overhang is also a factor. In the numerical simulation, the thermo-physical and mechanical properties are supposed to be isotropic but the microstructure and defects of the fabricated part, such as grain size, orientation, texture, [4,5,10,14] and porosities are also related to the scanning strategy. The texture of the overhang leads to the anisotropic behavior of the material, but the effect of anisotropic behavior on the residual stress and deformation of fabricated parts is still an unexplored area.

**Figure 12:**
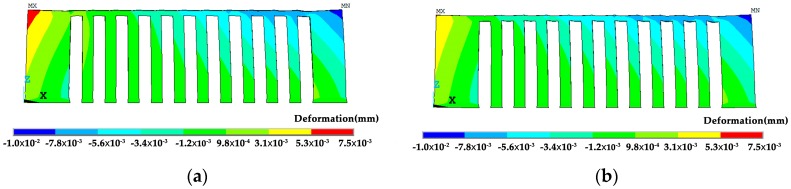
Shrinkage in the *x* direction of the specimens under different scanning strategies cooled to room temperature (deformation magnification 5×): (**a**) the *x* scanning route; and, (**b**) the *y* scanning route.

The shrinkage in the *x* direction of the corner is shown in Figure 12. The shrinkage of the left corner by the *x* scanning strategy is greater than the *y* scanning strategy. This concurs with the experimental results. 

**Figure 13:**
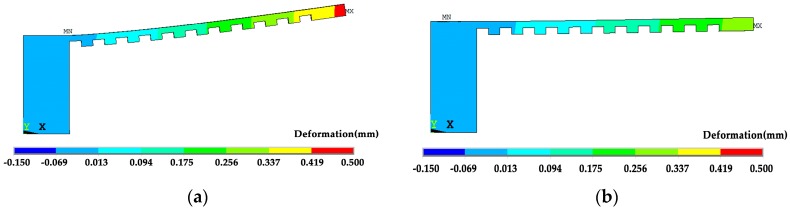
Deformation of the specimen under different scanning strategies as the supports and the far end are removed (deformation magnification 2×): (**a**) the *x* scanning route; and, (**b**) the *y* scanning route.

**Figure 14:**
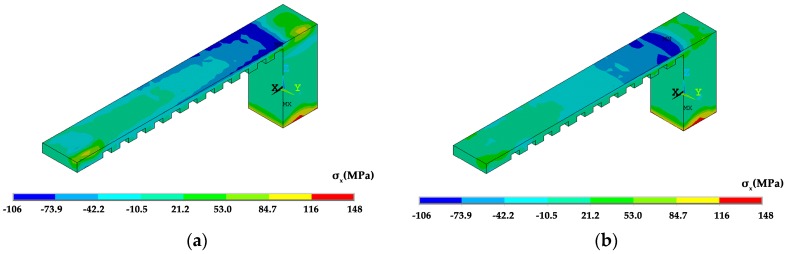
Residual stress σ_x_ of the specimen under different scanning strategies as the supports and the far end are removed: (**a**) the x scanning route; and, (**b**) the y scanning route.

As the constraints are removed the overhang bends upwards. However, the bending of the specimen by the *x* scanning route is 0.43 mm which is almost two times larger than that by the *y* scanning route of 0.26 mm, as shown in Figure 13. The calculated deformation tendency is the same as the experimental results, and the level is similar. These values are also affected by the number of layers and the size of the specimen. In comparison to Figure 10, the residual stress in the detached specimen is greatly reduced, as shown in Figure 14.

**Figure 15:**
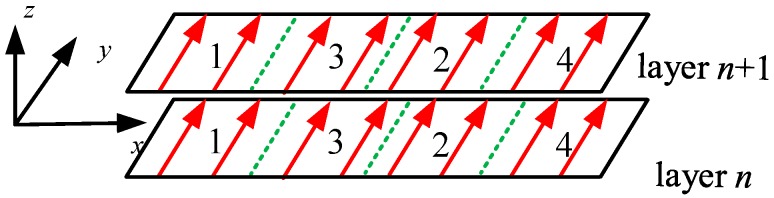
Discontinuous *y* scanning strategy by the sequence of blocks 1,2,3,4.

Therefore, it can be concluded that the residual stress along the *x* direction is the main course responsible for the contraction deformation. The *y* scanning pattern of the laser beam results in a relatively small heat affecting zone and, therefore, the contraction is limited to a small area which greatly contributes to the decrease of residual stress and deformation. To further decrease the deformation, the overhang can be divided into several blocks in the *x* direction. The scanning sequence of the blocks in the transverse direction is achieved by 1-2-3-4, as shown in Figure 15. This kind of discontinuous scanning will interrupt the contraction along the *x* direction and lead to less residual stress and deformation.

## 5. Conclusions

Different scanning routes for the overhang, including longitudinal direction, transverse direction, and alternating scanning routes, were studied by experiments and numerical simulation. The main results can be summarized as follows:

1. The longitudinal scanning resulted in a significant upward bending deformation of the overhang as it was cut from the baseplate. However, there was less deformation of the overhang as it was fabricated by the transverse and alternating scanning routes.
2. The longitudinal scanning made the temperature distribution of the entire overhang relatively uniform, which made the overhang contract simultaneously. However, the transverse scanning made the high temperature zone exist at the final scanning area, which made the overhang contract in the longitudinal direction at different time periods.
3. The longitudinal scanning resulted in significant residual stress on the overhang. However, the transverse scanning route significantly decreased the residual stress.
4. The transverse scanning route can avoid the simultaneous contraction along the entire overhang. The discontinuous transverse scanning route further reduced the residual stress and deformation of the overhang.

## Figures and Tables

**Table 1:** The chemical components of AlSi10Mg powder (wt %).

Al	Si	Mg	Fe	Mn	Ti	Zn	Cu	Ni
Balance	10.0	0.40	0.50	0.40	0.15	0.10	0.05	0.05

**Table 2:** Temperature-independent properties of AlSi10Mg alloy [22,25].

Temperature (K)	298	473	673	820	870	1000
Density (kg/m^3^)	2650	2550	2400	2200	2000	1900
Thermal conductivity (W/(m∙K))	147	159	159	159	100	105
Specific heat capacity (J/(kg∙K))	739	797	838	922	1100	1000
Density (powder) (kg/m^3^)	920	930	950	1000	-	-
Thermal conductivity (powder) (W/(m∙K))	1.5	1.6	1.7	1.8	-	-
Specific heat capacity (powder) (J/(kg∙K))	443	478	503	553	-	-
Young’s modulus (GPa)	69	67	62	53	41	30
Poisson’s ratio	0.33	0.33	0.33	0.33	0.33	0.4
Coefficient of thermal expansion (10^−6^/K)	21.7	22.5	23.5	23.3	25.5	25.5
Yield strength (MPa)	195	150	105	70	30	20
Plasticity hardening coefficient (GPa)	0.69	0.67	0.62	0.53	0.41	0.30
